# Left Atrial Septal Pouch (LASP) and Cryptogenic Stroke: A Narrative Review

**DOI:** 10.7759/cureus.64245

**Published:** 2024-07-10

**Authors:** Palwasha Farooqi, Adila Yaqobi, Bushra Mia Khail, Jose A Niño Medina, Zainab Obaid Ullah, Abed Saeed, Haroon Alamy, Syed Ahmad Farooqi, Najim Azizi, Leonor E Duarte, Torgot Ghani, Hasibullah Aminpoor

**Affiliations:** 1 Internal Medicine, Kabul University of Medical Sciences, Kabul, AFG; 2 Pediatric Medicine, Kabul University of Medical Sciences, Kabul, AFG; 3 Internal Medicine, Ali Abad Teaching Hospital, Kabul, AFG; 4 Law and Political Sciences/Health Sciences, University of Carabobo, Valencia, VEN; 5 Internal Medicine, Sir Ganga Ram Hospital, Lahore, PAK; 6 Cardiovascular Medicine, Ali Abad Teaching Hospital, Kabul, AFG; 7 Internal Medicine, Armed Forces Academy of Medical Sciences, Kabul, AFG; 8 Internal Medicine, Liaquat University of Medical and Health Sciences, Jamshoro, PAK; 9 Surgery, Herat Regional Hospital, Herat, AFG; 10 Medical Education, University of Carabobo, Valencia, VEN; 11 Internal Medicine, Wazir Mohammad Akbar Khan Hospital, Kabul, AFG

**Keywords:** mechanical thrombus, left atrial appendage occlusion, treatment of left atrial septal pouch, left atrial thrombus, stroke

## Abstract

Over 20% of ischemic strokes are cardioembolic strokes, necessitating research into thrombus formation locations, particularly the left atrial appendage (LAA). The left atrial septal pouch (LASP), which is linked to thrombus development and stasis, has drawn attention recently as a possible thromboembolic location, especially in atrial fibrillation (AF). The primary aim of this review is to explore LASP's role in cryptogenic strokes and to discuss the methods used to assess LAA anatomy. Imaging modalities such as cardiac computed tomography (CT) and transesophageal echocardiography (TEE) are crucial for diagnosing and characterizing LASP. LASP, found in about one-third of individuals, provides an additional site for thrombus development in the left atrium. The potential clinical implications of LASP-related thromboembolic events include the need for targeted therapeutic strategies, such as anticoagulant medication and, in some cases, consideration of LASP closure to prevent recurrent strokes. Further investigation is required to elucidate LASP's involvement in thromboembolic events and to guide stroke prevention in at-risk patients.

## Introduction and background

Over 20% of all ischemic strokes are cardioembolic strokes, where the left atrial appendage (LAA) is often investigated as the main site of thrombus development. Less is known about the underlying causes of thrombi that can also form in the left atrium (LA) outside of the appendage, particularly in rheumatic heart disease. The left atrial septal pouch (LASP) has been identified as a new atrial structure that may be linked to thromboembolic problems and stasis [1-2].

About one-third of people have LASP, which offers an additional location for thrombus development and stasis in the LA. Thrombi adhering to the septal LA have been identified in certain instances, indicating a possible function of LASP in thrombus development. Echocardiographic characteristics linked with LASP-related thrombi can be varied and include high filling pressures or dense spontaneous echo contrast (SEC) as a result of missing atrial contractions from atrial fibrillation [3]. LASP offers an additional location for thrombus development and stasis in the LA. A possible involvement of LASP in thrombus development has been suggested by the observation of thrombi adhering to the septal LA in certain instances. A variety of echocardiographic characteristics can be seen in patients with LASP-associated thrombi, such as high filling pressures or dense SEC, which is caused by missing atrial contractions from atrial fibrillation [4]. 

To clarify LASP's involvement in thromboembolic events and direct therapeutic approaches for stroke prevention in at-risk patients, more investigation is required. Despite its prevalence, the role of LASP in thrombus formation and stroke remains underexplored. Despite its prevalence, the exact role of LASP in thromboembolic events, such as cryptogenic strokes, remains unclear. This knowledge gap necessitates further investigation to clarify LASP's involvement in thromboembolic events and to guide therapeutic strategies for stroke prevention in at-risk patients. Further research should focus on understanding the conditions that promote thrombus formation in LASP, the effectiveness of different imaging techniques for identifying LASP-related thrombi, and the potential benefits of targeted treatments. Elucidating these aspects will help develop better strategies for stroke prevention and management in patients with LASP.

## Review

What is LASP?

A common anatomical variation known as the LASP occurs when the septum primum and septum secundum incompletely fuse at the interatrial septum [5-6]. This pouch can potentially raise the risk of blood clot formation, leading to systemic thromboembolism and ischemic stroke [7-9]. Diagnosis typically involves a transesophageal echocardiogram (TEE) with a bubble study. However, the clinical significance of LASP remains uncertain despite its potential involvement in cardioembolic stroke [10]. However, various studies are presented in this review that incline toward the involvement of LASP in thromboembolic events. 

Etiology

Interatrial septation develops throughout pregnancy, with the interatrial septum partially open during the embryonic stage, allowing direct blood flow from the right to the left atrium, bypassing the pulmonary circulation. This connection spontaneously closes shortly after birth due to increased left atrial blood pressure pushing the oval foramen's flap valve against the "secondary septum," an infolding of the atrial wall. Over time, micro-injuries and cicatrization of nearby structures lead to the slow closure of this connection, known as lifetime remodeling of the human interatrial septum [11]. Approximately 25% of adults do not experience this closure, potentially resulting in a shunt between the atria via the patent foramen ovale (PFO) channel. The septal pouch forms due to the partial union of the primary and secondary septum, with three possible configurations based on the location of fusion: left septal pouch, double septal pouch, and right septal pouch. In older individuals, increased adhesion, particularly in small pouches, leads to pouch closure and the development of a smooth septum [12]. For individuals with a fully fused oval foramen (short PFO channel), early-life closure occurs, resulting in a smooth septum. It is important to note that contrary to popular belief, the coexistence of PFO with septal pouches is not possible [13]. The septal pouch formed by the above-mentioned process has been implicated in developing the thrombi leading to thromboembolic events and strokes.

Mechanisms

The formation of thrombi in the LAA represents a critical concern in nonvalvular AF, driven by various factors that influence hemodynamic function, prothrombotic conditions, endothelial damage, and LAA morphology.

Up to 90% of instances of cardioembolic strokes are known to be caused by the LAA, especially in nonvalvular AF [14]. The thrombus development and consequent stroke risk linked to the LAA are caused by many factors:

Hemodynamic Function

The LAA's hemodynamic function determines the risk of thrombus formation, with various flow patterns having an impact on the incidence of thrombus formation. Spontaneous echo contrast and thrombus development are most common in type III flow patterns, which are distinguished by the lack of active emptying during AF [15]. Reduced LAA peak flow velocity is thought to be a very reliable independent indicator of a higher risk of thromboembolism [16].

Prothrombotic Condition in AF

Higher blood levels of coagulation activity-related indicators are indicative of a prothrombotic and hypercoagulable condition in AF [17].

Endothelial Damage

AF causes inflammation, fibrosis, and endothelium damage in the left atrium, especially in the LAA [18].

LAA Size and Shape

More intricately shaped LAAs are linked to an increased risk of thrombus development and spontaneous echo contrast [19].

Association between LASP and cryptogenic stroke

The following studies indicate a potential link between LASP and cryptogenic stroke, although findings across studies show some variability.

A recent meta-analysis revealed that LASP is found in 33.1% of cryptogenic stroke cases, significantly more than in non-cryptogenic strokes at 20.6%. The odds of a cryptogenic stroke are 1.618 times higher in individuals with LASP (p < 0.001). This highlights LASP as a key risk factor for cryptogenic strokes, underscoring the need for additional research to establish management and prevention strategies [20].

A recent case report was presented with a case of recurrent thromboembolic conditions in a patient with normal sinus rhythm and no other cause of thrombus other than LASP [21]. One case-control study reported prevalence rates of LSP in stroke patients and control individuals (28.9% vs. 29.3%; p = 0.93). Specifically, in the subgroup of patients with cryptogenic stroke, the prevalence was comparable to controls (31.9% vs. 29.3%; p = 0.70). The multivariable analysis did not find an association between LSP and ischemic stroke (odds ratio (OR) 1.09, 95% confidence interval (CI) 0.64 to 1.85) or cryptogenic stroke (OR 1.41, 95% CI 0.71 to 2.78) [22].

In a retrospective study of 1,126 patients, 176 had LASP. Of these, 32 (21.6%) were in the ESUS+ (embolic strokes of undetermined source) group and 144 (14.7%) in the ESUS- group. Multivariate analysis indicated an independent correlation between LASP and ESUS (p = 0.019). Notably, 61.9% of LASPs were not reported in initial TEE reports [23].

Another observational study found that 31% of individuals had LASPs, with a prevalence of 28% in stroke patients, 25% in non-cryptogenic stroke patients, and 43% in cryptogenic stroke patients. The cryptogenic stroke subgroup had a significantly higher LASP prevalence compared to the non-cryptogenic subgroup (p = 0.02), suggesting LASP as a potential stroke risk factor [24].

A study involving 324 patients undergoing TEE identified LASPs in 98 participants (59 LASP and 40 RASP). LASPs were significantly deeper than right atrial septal pouches (RASPs) (10.1 ± 5.2 mm vs. 4.4 ± 1.4 mm; p < 0.0001). While there were no significant differences in patient characteristics, ischemic stroke was more common in patients with LASPs (21 vs. 10 individuals). Both univariable (OR = 2.43, 95% CI = 1.1-5.5, p = 0.033) and multiple logistic regression models (OR = 2.45, 95% CI 1.1-5.8, p = 0.036) confirmed the association between LASP and increased ischemic stroke risk [25].

Collectively, these studies highlight a potential link between LASP and cryptogenic stroke, although the strength of the association varies. Meta-analyses and certain observational studies suggest a significant correlation, while the case-control study does not find a strong link. The evidence leans toward LASP being a risk factor for cryptogenic stroke, but further research is needed to clarify the mechanisms and strengthen the causal inference.

Imaging modalities

Role of TEE in LAA Exclusion

Percutaneous exclusion of the LAA is a developing strategy to prevent embolic events in patients with nonvalvular AF. Several percutaneous devices have been introduced to isolate the LAA from systemic circulation. Two- and three-dimensional TEE is crucial in assessing LAA anatomy, guiding device selection and implantation procedures, and evaluating device effectiveness post-implantation. Studies suggest that three-dimensional (3D) TEE offers superior visualization compared to 2D TEE [26].

Visualizing LASP

Two methods are typically used to visualize the LASP in vivo: TEE and contrast-enhanced electrocardiogram-gated multi-slice computed tomography (MSCT) of the heart. The accuracy of these methods in detecting LASPs has not been fully determined. For MSCT, a 64-row dual-source scanner was used, with a contrast agent administered intravenously. Imaging parameters included a tube voltage of 100-120 kV, an effective tube current of 350-400 mA, and collimation of 2 × 32 × 0.6 mm. Post-processing and evaluation were performed using dedicated software. For TEE, examinations were conducted with a 2D ultrasound system, and the interatrial septum was assessed in specific views without the Valsalva maneuver or saline contrast administration. Post-processing and evaluation were conducted using dedicated software [26,27].

Comparative Studies

In a study comparing preprocedural 2D-TEE and pre- and post-procedural MSCT, successful LAA closure (defined by no contrast leakage at three-month follow-up MSCT) was achieved in 87% of patients. MSCT-based sizing resulted in correct device selection in 83% of cases, significantly higher than the 57% accuracy achieved with 2D-TEE sizing (p < 0.01). These findings suggest that MSCT-based LAA closure device size selection is superior to conventional 2D-TEE-based sizing. The choice of sizing method depends on the device design [28].

Clinical relevance and practical applications

From the above studies, we can conclude that TEE, particularly 3D-TEE, is crucial for guiding device selection and implantation in LAA exclusion procedures. While 3D-TEE provides superior visualization, MSCT offers more accurate device sizing, improving the success rate of LAA closure. Understanding the strengths and limitations of each imaging modality helps clinicians choose the most appropriate method for each patient scenario, enhancing procedural outcomes and patient safety.

Management

Imaging and Diagnosis

Several considerations must be made while treating cryptogenic stroke associated with the LASP. Initially, TEE is often utilized for a thorough imaging assessment to detect and define LASP [29].

Medical Management

If LASP has been identified, anticoagulation therapy may be considered to reduce the risk of thromboembolic events. Before making this decision, which should take into consideration the patient's comorbidities, bleeding risk, and overall stroke risk profile, consult a neurologist or stroke expert [30-31]. Patients with LASP who had a cryptogenic stroke were treated for years with antiplatelet and hyperlipidemia medication; nevertheless, because of conflicting study findings, closure was not advised. Prior research such as CLOSURE I, PC, and RESPECT did not demonstrate the advantages of closure over medical treatment [30,31,32].

Lifestyle and Risk Factor Management

Furthermore, considering the link between LASP and a higher risk of cryptogenic stroke, lifestyle changes and the management of underlying risk factors such as diabetes, hypertension, and hyperlipidemia may be helpful preventative strategies [30,31].

Surgical Intervention

Closing the LASP may be an option for some individuals who experience repeated cryptogenic strokes even with the best medical care to stop further embolic occurrences. Nonetheless, the choice to move forward with closure should be unique and founded on a thorough evaluation of the advantages and disadvantages and the preferences of the patient [29,33]. According to a 2014 meta-analysis, pouch closure did not significantly lower the risk of recurrent stroke or TIA in the near term. New research and long-term follow-ups, such as those by Mas et al. and Søndergaard et al., have shown that there is a decreased risk of ischemic stroke and new brain infarctions following pouch closure, all without an increase in adverse events. Comparing pouch closure to medication therapy, De Rosa et al.'s meta-analysis found that pouch closure successfully avoids recurrent stroke and TIA. These results suggest a paradigm change in the treatment of pouch closure cryptogenic stroke [34-36].

Multidisciplinary Approach

The management of LASP-related cryptogenic stroke emphasizes the importance of individualized treatment decisions, considering patient preferences and overall health status. A multidisciplinary approach involving neurologists, cardiologists, and patient preferences is crucial to ensure optimal outcomes and personalized care.

Guidance of LAA closure through 3D printed cardiac reconstruction

A study by Pellegrino et al. discusses the utilization of 3D-printed models in guiding LAA closure procedures. This innovative technology aids clinicians in optimizing device placement and ensuring precise anatomical fitting [37].

Case descriptions

In the first case, a 3D-printed LAA model was used to position a Coherex Wavecrest device effectively within the LAA, ensuring proper compression and sealing. This precise modeling facilitated a successful outcome by allowing clinicians to rehearse the procedure and adjust the device placement as needed.

In the second case, the 3D model played a crucial role in accurately sizing the device, leading to the selection of a larger Amulet device for optimal coverage of the LAA vestibule. The model provided a detailed anatomical assessment, which traditional imaging techniques like angiography and TEE could not achieve as accurately [37].

Broader implications, key takeaways, and future uses

The report underscores the limitations of traditional imaging in accurately estimating LAA measurements and highlights the benefits of 3D-printed models for precise device sizing and procedural planning. These models enable detailed preoperative planning, improving LAA closure outcomes by allowing clinicians to select the appropriate device size and rehearse procedures. The future of 3D printing in cardiology includes its application in other complex procedures, offering personalized patient care and reducing procedural risks.

## Conclusions

The significance of the LASP's relationship with cryptogenic stroke underscores the need for its identification and treatment in stroke patients. Imaging modalities including cardiac CT and TEE are essential for both diagnosing and characterizing LASPs. After being diagnosed, cryptogenic stroke linked to LASP is managed using a multidisciplinary strategy that takes anticoagulant medication, lifestyle changes, and the management of underlying risk factors into account. Although antiplatelet and hyperlipidemia medication were the mainstays of previous therapies, new research indicates that some patients, especially those who have repeated cryptogenic strokes, may benefit from contemplating the closure of LASPs. The practical implications for clinical practice involve a personalized approach to treatment, considering the individual patient's risk factors, preferences, and overall health status. Implementing a multidisciplinary approach involving neurologists, cardiologists, and other healthcare professionals is crucial in optimizing patient outcomes. To clarify the best care practices and long-term results in this patient population, more investigation is necessary. Further research should focus on the long-term efficacy of LASP closure, optimal patient selection criteria, and the development of guidelines to standardize treatment protocols. This research will be vital in enhancing our understanding of LASP-related cryptogenic stroke and improving clinical outcomes through evidence-based practices.
